# Effect of incentives on insecticide-treated bed net use in sub-Saharan Africa: a cluster randomized trial in Madagascar

**DOI:** 10.1186/1475-2875-9-186

**Published:** 2010-06-27

**Authors:** Paul J Krezanoski, Alison B Comfort, Davidson H Hamer

**Affiliations:** 1 Boston University School of Medicine, Boston, MA 02118, USA; 2 Program in Health Policy, Harvard University, Cambridge, MA 02138, USA; 3 Center for Global Health and Development, Boston University School of Public Health, Boston, MA, USA; 4 Department of International Health, Boston University School of Public Health, Boston, MA, USA; 5 Section of Infectious Diseases, Boston University School of Medicine, Boston, MA, USA

## Abstract

**Background:**

Insecticide-treated bed nets (ITNs) have been shown to reduce morbidity and mortality due to malaria in sub-Saharan Africa. Strategies using incentives to increase ITN use could be more efficient than traditional distribution campaigns. To date, behavioural incentives have been studied mostly in developed countries. No study has yet looked at the effect of incentives on the use of ITNs. Reported here are the results of a cluster randomized controlled trial testing household-level incentives for ITN use following a free ITN distribution campaign in Madagascar.

**Methods:**

The study took place from July 2007 until February 2008. Twenty-one villages were randomized to either intervention or control clusters. Households in both clusters received a coupon redeemable for one ITN. After one month, intervention households received a bonus for ITN use, determined by visual confirmation of a mounted ITN. Data were collected at baseline, one month and six months. Both unadjusted and adjusted results, using cluster specific methods, are presented.

**Results:**

At baseline, 8.5% of households owned an ITN and 6% were observed to have a net mounted over a bed in the household. At one month, there were no differences in ownership between the intervention and control groups (99.5% vs. 99.4%), but net use was substantially higher in the intervention group (99% vs. 78%), with an adjusted risk ratio of 1.24 (95% CI: 1.10 to 1.40; p < 0.001). After six months, net ownership had decreased in the intervention compared to the control group (96.7% vs. 99.7%), with an adjusted risk ratio of 0.97 (p < 0.01). There was no difference between the groups in terms of ITN use at six months; however, intervention households were more likely to use a net that they owned (96% vs. 90%; p < 0.001).

**Conclusions:**

Household-level incentives have the potential to significantly increase the use of ITNs in target households in the immediate-term, but, over time, the use of ITNs is similar to households that did not receive incentives. Providing incentives for behaviour change is a promising tool that can complement traditional ITN distribution programmes and improve the effectiveness of ITN programmes in protecting vulnerable populations, especially in the short-term.

## Background

Insecticide-treated bed nets (ITNs) have been demonstrated to be of significant value in reducing morbidity and mortality due to malaria in sub-Saharan Africa[1,2]. Despite their accepted effectiveness, there remain barriers to the use of ITNs in vulnerable households, including both supply- and demand-side constraints, which make rapid scale-up of ITN coverage difficult to achieve. On the demand-side, much attention has been paid to the need for subsidization of the cost of ITNs as a means to increase ITN coverage, because cost has been identified as an important barrier to ITN ownership[3,4].

Once households acquire an ITN, there are still significant questions about the determinants of actual use[5,6]. Reports of misuse of subsidized nets complicate the efforts of government and non-governmental organization (NGO) malaria prevention programmes seeking to promote ITN coverage and use in poor countries[7,8]. Various studies have demonstrated that providing nets for free is important to overcome the cost barriers associated with obtaining an ITN [9,10], but mere provision of free ITNs may not be enough to provide coverage for the required 80% of households that the WHO has indicated is necessary to provide community-wide "mass" protection from malaria[11-13].

Interventions to increase the actual use of ITNs have focused on subsidizing the costs associated with net ownership and providing education about malaria transmission and prevention. There are other tools which could be used to promote healthy behaviours, however, given evidence of the effectiveness of performance-based incentives in encouraging healthy behaviours such as weight loss and tobacco cessation[14,15]. Only in recent years have performance-based incentives been more widely applied to the promotion of behaviours related to health problems in developing countries[16], such as childhood immunizations[17], nutrition[18], maternal care[19] and tuberculosis detection and treatment[20].

To date, there have been relatively few randomized controlled trials looking at the effects of incentives on health behaviours in poor countries and no study has yet looked at the effect of incentives on the use of ITNs. Given the challenge of achieving high coverage rates of ITNs, even after their free distribution, reported here are the results of a village-clustered randomized controlled trial which provided household-level incentives in order to stimulate ITN use following a free bed net distribution campaign in Madagascar.

## Methods

### Study site and population

The study took place in rural villages located around the town of Ambalavao in the Ambalavao district of Madagascar. This location was chosen because of previous relationships with local partners developed by two of the authors (PJK and ABC) during service in the Peace Corps from 2003 to 2005. Madagascar is among the poorest countries in the world, with 85% of the population living below the poverty line, defined as $2 a day[21]. The Ambalavao district (population of 210,000; 5,000 km^2^) is comprised of the large town of Ambalavao (population of 30,000) in addition to scores of rural villages ranging from ten to seventy households clustered around land used for rice farming and cattle grazing.

The inhabitants of these rural villages belong primarily to the Betsileo ethnic group. There are 18 ethnic groups in Madagascar, organized roughly into two groups: the central highland peoples with their Indonesian and Asian origins and the more traditionally African origin of the coastal people. The Betsileo are of the former group, making up roughly 12% of the population of Madagascar. They have a strong rural tradition of rice farming, building terraces along the slopes of the steep hills of the central highlands[22].

The district of Ambalavao is located in a valley abutting the eastern tropical forest in the south central part of the country, with an average elevation of 800 metres descending from the central highlands to the southern plateau. The study was carried out during six months from July 2007 until February 2008, beginning just after the rice-harvesting season, continuing through the dry season and finishing in the rainy season.

### Malaria in Madagascar

Like the rest of sub-Saharan Africa, malaria is a public health challenge for the country of Madagascar. Malaria accounts for 16% of all outpatient visits in Madagascar. It is the leading cause of child mortality and the second leading cause of death for all age groups. Malaria kills nearly 20,000 children a year under five years of age and accounts for 11% of all deaths and 15% of years of life lost [23,24]. The entire country is considered to be at risk for endemic malaria, while some areas of the central highlands are vulnerable to epidemics with seasonal transmission from September to June. All four species of malaria parasite are endemic in Madagascar with the majority of infections caused by *Plasmodium falciparum *and approximately 10% caused by *Plasmodium vivax *and other species[25]. In 2007, with the support of the Global Fund for AIDS, Tuberculosis and Malaria, the country's National Malaria Control Programme put into place a five year plan with the goal of providing free ITNs for all pregnant women and children under five years of age. However, based on 2003-2006 estimates, only 35% of children under five in Madagascar were sleeping under a bed net of some kind and no children were sleeping under ITNs[26].

### Malaria characteristics of Ambalavao district

With an average elevation below 1,000 meters and average temperatures of 20°C, malaria transmission in the Ambalavao district is considered to be stable year round. A comparison of presumed monthly malaria cases to annual cases shows that the majority of transmission occurs during the rainy season from January to April. The primary vectors are *Anopheles funestus *and *Anopheles arabiensis *with *Plasmodium falciparum *as the primary parasite[27].

In 2002, one out of every five health care visits in the Ambalavao district was for presumed malaria[21]. Much of the baseline malaria characteristics of the Ambalavao district must be estimated from statistics at the provincial level. A study conducted in 2004 found that, in the Fianarantsoa province, which includes the district of Ambalavao, 11% of children under five years of age reported a fever in the previous two weeks[28]. While data on ITN use in the Ambalavao district are not available, in 2008, 34% of households in the Fianarantsoa province owned a net and 30% of children under five years of age and 34% of pregnant women reported sleeping under a net the night before.

### Randomization procedures

The study was conducted in twenty-one villages located within five kilometers of the central town of Ambalavao. The study participants included each household in the study villages. Randomization was performed at the village level, with each village representing a cluster. This cluster design was used to increase acceptability of the intervention and decrease potential programme backlash by study participants if some households living in a village received bonuses while others did not. Initially, a total of eighty villages were identified within five kilometers of Ambalavao using a local map, with twenty villages coming from each of the four quadrants according to their location relative to Ambalavao (i.e. northeast, northwest, southeast and southwest). The villages were listed alphabetically by quadrant and numbered sequentially from 1 to 80. This listing by quadrant ensured a reasonably balanced sampling of villages from all four quadrants. A random number was then chosen by a throw of dice. Twenty-one villages were then selected from the list of eighty by counting off the names of the villages using the random number and looping back through the list until all 21 villages were chosen. The names of the villages were then written on slips of paper and drawn randomly from a hat. The first eleven villages drawn were allocated to the control group and the next ten were allocated to the intervention group.

### Study procedures

#### Surveyor training

A team of 15 health workers was hired to undertake village outreach and perform the study surveys. The health workers were members of two Malagasy non-governmental associations, Association Fanilo and Association Avotra, a men's and women's association respectively. These two organizations were created with the assistance of two of the authors (PJK and ABC) during their time as Peace Corps Volunteers in Ambalavao from 2003 to 2005. In 2002, John Snow International (JSI) developed a network of health workers throughout Madagascar, and many of the health workers had received training as part of this effort. The health workers had received additional training during their work with the authors from 2003 to 2005, and also during their work on a previous ITN study based in Ambalavao in 2004. This previous ITN study was performed in different villages from the current study. For the current study, the surveyors received a full day of training which included a description of the study, a module on ethics and obtaining informed consent from study subjects, a review of the survey instruments and practical experience performing the surveys on two test subjects.

#### Protocol visit

Following the training, health workers were then dispatched to each village for a "protocol visit" where the project was introduced to the local village leader. This visit also allowed the surveyors to arrange for a convenient time to return to the village when at least one adult member of each household would be available for the baseline survey.

#### Baseline survey and coupon distribution

Following the initial protocol visit, the survey teams were sent to perform the baseline survey and enroll study households. A "household" was defined as a family unit or individuals living separately from any other family unit or individuals (i.e. living spaces could not be contiguous). Criteria for inclusion in the study encompassed all households located within one of the study villages. Households who had no member present during the baseline survey were able to enroll during the net distribution week, if a village leader could attest to their residence in the study village. There were only three households who took advantage of this enrollment option. At the baseline encounter, each household was surveyed on demographic characteristics, household assets, perceptions of risk related to malaria and baseline ITN ownership and use. In addition to asking whether a net was used the night before, surveyors also entered each household and assessed visually whether or not a net was mounted over a bed. In this study, because of potential inconsistencies from self-reported net use, the mounting of a net over a bed is used as a proxy for net use and will be referred to interchangeably. Households were unaware of when the survey personnel would arrive to inspect the household. In the survey questions about bed net ownership and use, no distinction was made between insecticide-treated and untreated bed nets. This does not change the underlying assumption that bed net coverage is insecticide-treated bed net coverage, because the only bed nets on the market in the Ambalavao area during this time were ITNs. In addition, all of the households who reported bed net ownership at the baseline survey reported obtaining their nets through ITN distribution programmes at local health clinics or through community-based health educators who are provided by government programmes with ITNs for resale. At the baseline encounter, each household was also given a coupon redeemable for one free ITN and instructions on how to redeem the coupon. Additionally, in the intervention villages, the households were informed that they would receive a bonus if they were using their nets when the survey team returned at some random time in the coming month. The value and form of the bonus was not disclosed at that time; instead, the households were informed that they would receive a "prize" for correct use of the free ITNs.

#### ITN description

The ITNs used in the study were obtained through Population Services International (PSI), the primary provider of ITNs in the region. The ITNs were white and rectangular shaped Permanet brand (Vestergaard-Frandsen, Denmark) long-lasting insecticide-impregnated nets (LLINs) made of polyester. The dimensions were 150 × 180 × 190 cm. These LLINs are expected to last up to three years or twenty-one washings. The ITNs were packaged in plastic along with string and instructions in the local language explaining how to mount the net over a bed.

#### ITN distribution

The coupons distributed at the baseline survey were redeemable in the central town of Ambalavao. In the interest of convenience for study participants, ITN distribution was begun on the local market day when many villagers typically come to Ambalavao. In addition to the instructions given during the baseline survey, radio announcements were made and signs were created to direct the study participants to the location where they could redeem their coupons. The ITN distribution site was on a major road off the town center located in an empty lot between some shops. The study team sat with the ITNs under a covered area and a queue was created. Coupon holders were required to hand over one coupon in exchange for one ITN. There was no limit on the number of coupons an individual could redeem. Following the exchange, the coupon number was registered and the coupon was destroyed. Nearly all of the ITNs (~95%) were distributed on the initial market day, but coupons were honoured for the next week.

#### One month surveys and bonus distribution

One month after the baseline survey, survey teams returned to all households for the one month follow-up surveys. Households were asked whether they owned a net and were using the net. The surveyors then visually inspected whether an ITN was mounted over a bed. In the intervention villages, households who had correctly mounted an ITN over a bed were given the bonus. The bonus consisted of 2,000 ariary (approximately $1) worth of household goods packaged in a plastic bag: coffee, sugar, salt, soap and rice.

#### Six month surveys

In order to determine the medium term use of nets and to try to capture seasonal differences in use patterns, the survey teams returned to all households at six months following the intervention to perform the final follow-up survey. At this encounter, households were again surveyed on perceptions of risk related to malaria, net ownership and net use. Surveyors performed visual inspections of sleeping areas to determine actual use of ITNs. The goal of the study was to stimulate early uptake and use of the ITNs with a one-time provision of incentives at the one-month visit, so no bonuses were promised at the one-month encounter for nets mounted at six months and no bonuses were provided at the six-month visit.

### Statistical analysis

#### Study endpoints

The primary outcomes were household ITN ownership and use one month following net distribution. The secondary outcomes were ITN ownership and use at six months following net distribution.

#### Power calculation

It was hypothesized that at one month post-intervention, 70% of households in the intervention villages would use their ITNs in comparison to 30% of the households living in the control villages. Initially, a two-armed study was envisioned, including 24 villages, with 12 villages in each arm, a 1:1 allocation of intervention to control villages, about 22 households per village and a total study population of 530 households. The unit of analysis in the study was at the household level within each village, meaning that sample size calculations were required to take into account possible correlation between the households in a village. Since information was not available on the intracluster coefficient (ICC) for the outcomes in this study, a rather large ICC of 0.25 was assumed. Based upon these assumptions, the study would need to include at least nine villages per arm with four households per village to have 80% power to show a difference in ITN use of 40% between the treatment and control groups.

#### Data management and analysis

Surveys were monitored on returning from the field for completeness and accuracy and surveyors were sent back to the field if data were missing. The collected data were then entered by members of the study team into Epi Info 3 (Centers for Disease Control and Prevention, Atlanta, Georgia) using a predefined template. Consistency of the data was ensured by random checks matching paper surveys to entered data. The data were cleaned following the completion of data entry and a master data set was created in Stata 10 (Statacorp, College Rd, TX) for analysis.

#### Description of t-tests and regressions

Both unadjusted and two forms of adjusted results are presented. These three sets of results represent three levels of adjustment for confounding variables. The crude and unadjusted results include comparisons of proportions of ITN ownership and use in the intervention and control groups at the baseline, one-month and six-month surveys. All adjusted results use cluster specific methods to account for randomization of households at the village (cluster) level as recommended in the latest CONSORT statement on the best practices for reporting the results of cluster randomized trials[29]. The first set of adjusted results from cluster-adjusted t-tests includes ICC coefficients and adjusted chi-squared values for the outcomes of interest. The second set of adjusted results is from a modified Poisson regression model, using a robust error variance, which is used to estimate risk ratios and p-values[30]. These regression results estimate the effects of the intervention while controlling for household and village level potential confounders such as household demographics, number of children under five years of age, number of pregnant women, household assets such as cell phones and cattle, cooking fuel type, home composition, number of rooms, number of beds, distance from water source, reported fevers in the previous month, reported deaths due to fever in the previous year, previous ITN ownership and use, village size and village location. A p-value of less than 0.05 was considered to be significant for all tests.

### Ethical clearance

Ethical clearance for the study was provided by the Boston University Medical Campus Institutional Review Board. Additional administrative approval was provided by the mayor of the town of Ambalavao, responsible for the villages in the district, and the Medicin Inspecteur of the Ambalavao health district, the local official in charge of all health-related activities in the district. Additionally, the chiefs of each village gave their approval for the study to take place in their village. Study participants provided verbal consent at the time of the surveys and coupons for the free ITNs were provided to all households in the study villages irrespective of whether or not they consented to participate in the study.

## Results

### Participant flow

**Figure 1:**
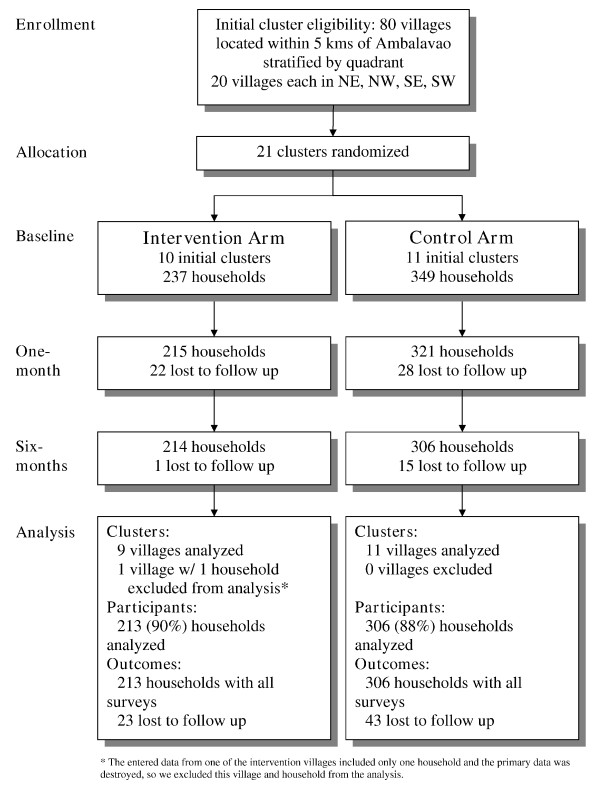
**Flow diagram for clusters and participants**. Diagram depicting the flow of clusters and participants in the study from participant enrollment through data analysis.

In July 2007, 21 villages were randomized to either the intervention or control arm of the study (Figure 1). This resulted in 10 clusters with 237 households in the intervention arm and 11 clusters with 349 households in the control arm, making a total of 586 participating households. Only 21 villages, rather than the planned 24, were randomized due to logistical convenience. This lower number was deemed acceptable because the mean number of households per village (~34) was substantially higher than what was required according to the power calculation (~22). By the time of data analysis, it was observed that the data from one village included information for only one household and the original surveys were mistakenly destroyed. This village was excluded from the analysis, reducing the intervention arm to 9 clusters vs. 11 clusters for the control arm. The overall loss to follow up rate was similar for both arms over the six-month study (10% in the intervention group vs. 12% in the control group).

### Baseline characteristics

A summary of the baseline characteristics of the study households shows that the households had a mean of five household members of which 53% were females (Table 1). Sixty-one percent of households had at least one child under five years old and 5% had a pregnant woman residing there. The majority of households had a home constructed of mud bricks (99%), a dirt floor (90%) and a thatch roof (78%). Sixty-five percent of households used an open water source for their daily water needs, a sign of lower socio-economic status and a potential source of increased risk of malaria infection. The households were an average of 9 minutes walk from their water source. At baseline, about 8% of households owned an ITN and about 6% were observed to have a net mounted over a bed in the household.

**Table 1:** Baseline characteristics of villages and households

Village Characteristics	Intervention	Control
	(9 villages; n = 213)	(11 villages; n = 306)
Number of households per village (mean ± SD)	34.7 ± 24.3	33.8 ± 17.3
	range: 9 - 74	range: 11 - 66
Households using open water source (%)	66.2	64.1
Village distance to open water source (mins) (mean ± SD)	139 ± 7.4	13.5 ± 7.1

**Household Demographic Characteristics**		

Number of members per household (mean ± SD)	5.2 ± 2.9	5.2 ± 2.6
	range: 1 - 20	range: 1 - 16
Number of men per household (mean ± SD)	2.4 ± 1.7	2.4 ± 1.7
Number of women per household (mean ± SD)	2.7 ± 1.6	2.7 ± 1.7
Number of children under five per household (mean ± SD)	0.92 ± 0.96	0.95 ± 0.89
	range: 0 - 5	range: 0 - 3
Households with at least one child under five (%)	57.7	61.4
Number of pregnant women per household (mean ± SD)	0.06 ± 0.25	0.05 ± 0.22
	range: 0 - 2	range: 0 - 1
Households with at least one pregnant woman (%)	5.2	5.2

**Malaria Prevention Characteristics**		

Baseline net ownership (%)	8.5	8.5
Baseline net use (%)	6.6	5.6
Baseline net mounted (%)	6.1	5.6
Children under five years using a net the night before (No (%))	11/18 (61)	12/26 (46)
Pregnant women using a net the night before (No (%))	18/18 (100)	25/26 (96)

**Malaria Risk Characteristics**		

Households reporting member with fever in last month (%)	42.3*	28.1
Number of households reporting death last year due to fever	0	1

* p-value < 0.01		

None of the village, household or malaria prevention characteristics differed significantly between the intervention and control groups, except for a greater proportion of the intervention households reporting a household member with a fever in the previous month compared with the control households (42% vs. 28%, p < 0.01). At baseline, there were no differences between the intervention and control group in the proportion of households owning a mosquito net, nor in the proportion with an ITN mounted over a bed in the household.

### One month outcomes

Both the intervention and control groups sought and obtained ownership of an ITN to an equal degree, with no differences in one-month ownership between the two groups (Table 2). There was, however, a large difference between the two groups in terms of overall net usage, with 99% of the intervention group versus 78% of the control group having a net mounted in their household at one month (p < 0.001). Adjusting for the clustered design and controlling for possible confounding factors resulted in an adjusted risk ratio for ITN use at one month in the intervention group of 1.24 (1.10 to 1.40; p < 0.001).

**Table 2:** Insecticide-treated bed net ownership and use at baseline, one month and six months

	Intervention No. (%)	Control No. (%)	Intracluster correlation coefficient	Cluster-adjusted χ² value	Adjusted risk ratio (95% CI)	Adjusted P value
Number of villages	9	11				
Number of households	213	306				

**Baseline**						

Net Ownership	18/213 (8.5)	26/306 (8.5)				
Net Mounted	13/213 (6.0)	17/306 (5.6)				

**One Month**						

Net Ownership	212/213 (99.5)	304/306 (99.4)	0.0231	0.05	1.00 (0.99 to 1.01)	0.79
Net Mounted	211/213 (99.1)	240/306 (78.4)	0.1894	5.23	1.24 (1.10 to 1.40)	<0.001

**Six Months**						

Net Ownership	206/213 (96.7)	305/306 (99.7)	-0.0007	5.26	0.97 (0.95 to 0.99)	<0.01
Net Mounted	198/213 (93.0)	274/306 (89.5)	0.0317	0.9	1.01 (0.96 to 1.08)	0.18

### Six month outcomes

**Figure 2:**
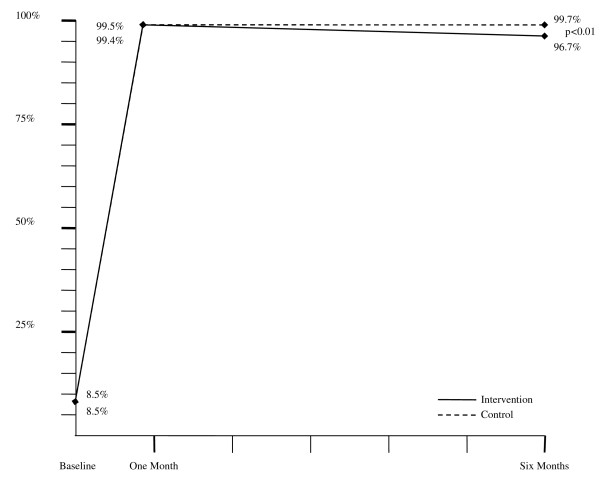
**Proportion of households owning an insecticide-treated bed net (ITN)**. Graphical depiction of the proportion of households in the control (dotted line) and intervention (solid line) groups which owned an ITN at baseline, one month and six months.

After six months, net ownership had slightly decreased in the intervention compared to the control households (Figure 2). Among households that had reported owning a net at one month, 3.3% of intervention households versus 0.3% of households in the control group no longer owned an ITN by six months (p < 0.01). This result was confirmed after adjusting for the clustered design and controlling for confounding factors, resulting in an adjusted risk ratio for net ownership at six months in the intervention group of 0.97 (0.95 to 0.99; p < 0.01).

**Figure 3:**
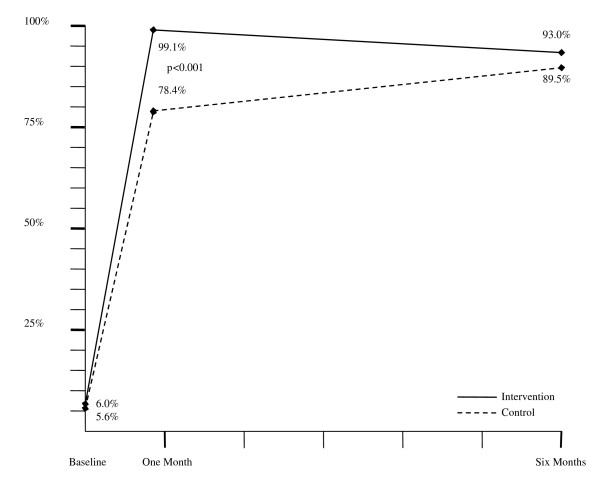
**Proportion of households with a mounted insecticide-treated bed net (ITN)**. Graphical depiction of the proportion of households in the control (dotted line) and intervention (solid line) groups which had an ITN mounted at baseline, one month and six months.

At six months there was a decrease in net use in intervention households, from 99% to 93%, and an increase in use in control households, from 78% to 89.5% (Figure 3). Restricting the analysis to only households that owned a net at six months, intervention households were much more likely to use their ITN than control households (96% versus 90%; p < 0.001).

## Discussion

The primary goal of this study was to test the effects of household-level incentives on the uptake and use of ITNs in a rural African setting. This study provides useful information regarding health behaviours in regards to ITNs, an increasingly important topic in development literature as the field progresses from elucidating the barriers to ownership to honing in on the factors that predict actual ITN use. In addition, as the first study analysing the effect of incentives on the use of ITNs in resource-poor settings, this study helps to link theories of behavioural economics to malaria prevention in developing countries. This is an important link, because, to this point, much of the literature about incentives for changing health behaviours has been limited to developed settings.

The main finding of this study is that provision of incentives for the use of ITNs increases the probability of ITN use by 24% in the immediate term. This increase in ITN use at one month occurred even though ownership of ITNs was essentially equivalent between the intervention and control households, highlighting the importance of looking at demand for ITN ownership and actual ITN use as interrelated, but separate, outcomes.

Eventual use of ITNs is a two-step process that involves, first, acquiring an ITN and, second, actual mounting and daily use of that ITN. Each of these steps is associated with barriers that stand in the way of prevention programmes whose goal is to maximize widespread use of ITNs and reduce malaria transmission. Much of the current literature, embodied in the debate over whether or not to distribute ITNs for free, focuses on the first step in this process, namely, the financial costs to households and other logistical barriers associated with the procurement and distribution of ITNs[4,5]. Lately, it has become clear that "closing the gap" between ITN ownership and use, especially among vulnerable populations[31], is increasingly important. For example, in many locales, there are differences between the local population's perceived risk of malaria and their actual risk. This discrepancy can result in poor adherence to ITN use during periods of high malaria transmission[32].

The present study had as one of its goals the elimination of barriers to ITN ownership, so that the behavioural impact of the incentive for ITN use could be isolated. This goal was achieved through the provision of ITNs for free to all households and the distribution of the ITNs through a centralized location equally accessible to all households. Since nearly 99% of both the intervention and control households owned ITNs at one month, this strategy was effective in getting the nets to the study households without bias, allowing for an isolation of the effects of the incentives on household behaviour in relation to ITN use.

Studies looking at the effect of incentives on changing health behaviours have found that incentives are effective in the short-term, but often fail to influence long-term behaviour[10]. This study demonstrates a similar result, with the intervention group decreasing its ITN use from 99% at one month to 93% by six months, presumably in response to the removal of the incentive for use. Other studies have shown that household motivation for use of ITNs, even in a free distribution programme with an education component, is dampened over time and depends on factors such as convenience and conceptions of malaria risk[33]. In this study, however, the control and intervention groups moved in opposite directions over time, as illustrated by Figure 3. While the intervention group slightly decreased its ITN use from one to six months, the control group greatly increased its ITN use from 78% up to 90%.

Accounting for this increase in six-month ITN use in the control group is easier than accounting for the decrease, albeit small, in the intervention group. The ITNs were originally distributed during the dry rice-harvesting season. When ITN use was evaluated six months later, it was during the rainy season. Thus, the increase in use by the control households could be due to seasonal changes when presumably there is greater mosquito burden and perceived need for ITNs. Still, since seasonal changes should affect both control and intervention groups equally, it is not clear why ITN use should increase so dramatically in the control households while slightly decreasing in the intervention households.

One possible explanation for this decrease in use in the intervention households from one to six months could be that the incentive artificially induced some households which do not intrinsically value ITNs to mount their nets at one month. By the time of the six-month survey, once the incentive had been removed, these households were no longer using their ITNs. The existence of even a small proportion of these types of households, which do not intrinsically value ITNs, could account for the evidence in other settings showing the divergence of ITNs towards unintended uses[8] and may play a role in the debate regarding the potential waste of ITNs when they are provided for free to poor households.

It is interesting to note, especially from the standpoint of behavioural economics, that inclusion in the intervention group made a household that owned an ITN at one month slightly less likely to own an ITN by six months. In fact, this decrease in ownership among incentivized households accounts for the bulk of the decrease in ITN use by six months. Behavioural economics predicts that the provision of incentives as an extrinsic source of motivation for behaviours can inhibit intrinsic motivation for use [34]. In addition, economic theory suggests that a negative price (i.e. paying a consumer to use a good in the form of an incentive) may provide a negative quality signal and potentially devalue the good in the view of some consumers. In the present study, conclusions are difficult to draw since the number of incentive households that gave up their net is small (~3%). Economic theory assumes that households should rationally demand preventative health goods, but our results suggest that more research needs to be done to look at the variable effects of incentives on valuing of these goods in poor households.

Despite the bonus' negative effect on ITN ownership after six months in a small portion of households, the majority of the intervention households continued to own a net after six months, thus demonstrating that they valued that net by using it. Although overall net use dropped from 99% to 93% in the intervention group from one month to six months, net use conditional on ownership remained high at 96% versus 90% in the control group. This means that intervention households that owned a net were significantly more likely to use their net than control households that owned a net. This may indicate that the bonus for mounting a net had a lingering effect on a household's use of a net, perhaps by creating a barrier in terms of effort to un-mount an already mounted ITN in some households.

These findings are consistent with past studies which demonstrated that the size of the incentive may not be correlated with an expected economic response[35,36]. In this study, the intervention households were promised a bonus of an indeterminate amount for ITN use at one month. The subsequent uptake and use of the ITNs in the intervention group was over 99%. Since the incentive was for an indeterminate amount, it appears that the mere offer of a bonus, of any value, was enough to overcome barriers to use. This finding is in line with previous studies of incentives which have found that the ability to overcome the psychological barrier to behaviour change is not necessarily proportional to the size of the incentive[10].

There are advantages and disadvantages to using incentives for boosting ITN use. In terms of advantages, the incentives themselves do not have to be of any definite value and they can be used to quickly and definitively cover almost an entire target group in a short amount of time. In contrast, there are two reasons why, as a practical matter, using incentives to boost ITN use on a larger scale may prove problematic. First, there are personnel and resource requirements associated with the follow-up visits required for the implementation of an incentive scheme. Secondly, incentives appear to only boost use of ITNs for a short period of time, and so repeat incentives may be required to sustain high level ITN use.

Despite these disadvantages, providing incentives for the use of ITNs could be a powerful tool, especially for programmes seeking coverage of vulnerable groups such as children younger than five years and pregnant women. There are a variety of methods, like social marketing and subsidization of ITN prices, that have been used to increase household ownership of ITNs. However, as mentioned previously, increasing household ownership is only the first step in achieving wider ITN coverage.

Once ITNs have made it into the household, education campaigns have traditionally been the tool of choice for addressing the gaps between ownership and use of ITNs in these groups[37]. Different types of education campaigns have been utilized, including mass communication, communications targeted to specific communities, schools, and health facilities and follow-up visits to households[38]. These education campaigns have all been based on the assumption that providing information about the risks associated with malaria and the mechanism of protection offered by ITNs will lead to higher perceived value of ITNs and thus increased use of ITNs. Education campaigns, however, may not be the most efficient means of ensuring ITN coverage of priority groups.

A possibly more cost-effective approach could be to use incentives to cover vulnerable populations during epidemics of malaria when malaria morbidity and mortality are high. Incentives and free ITNs could be provided for coverage of households with children under five or pregnant women. This intervention could quickly lead to the required 80% of households that the WHO has indicated is necessary to provide community-wide "mass" protection from malaria[11]. In addition, this targeted intervention would have the benefit of definitively covering the most vulnerable groups. Targeted approaches like the one presented here offer the opportunity to bypass the gap between ITN ownership and use and put direct control over who uses ITNs in the hands of policy makers and public health experts.

One limitation of this study is the short time period of the intervention. The study was not powered nor designed to look at longer term outcomes. It would be useful to know the outcomes after one year to see if the proportion of households using ITNs continued to converge over time, or if ITN use dropped off in both groups as is suggested by some other longitudinal studies[27]. Furthermore, this study was not designed to provide definitive information about how the presence of children under five years or pregnant women in the household affects a household's likelihood of using a net.

There is some potential discrepancy between using the observation of a mounted net over a sleeping area as an indicator of use, since some households have been found to not use nets in hot weather (e.g. an ITN is mounted above a bed but is not unfurled around the bed at night). Observed net mounting is a more reliable proxy than relying solely on self-reported use. In addition, direct observation of the household for a mounted net was performed during household surveys, which occurred at an unannounced time, so misleading the data collection team would have been difficult. Night-time visits and other methods of determining actual ITN use were not deemed logistically feasible. Another potential issue is that there was no distinction made between whether nets owned at baseline were insecticide-treated or not. However, as discussed, most households in this region did not have access to non-insecticide treated bed nets.

## Conclusion

The provision of behavioural incentives to households in rural Madagascar significantly increased the use of ITNs after one month compared to households that did not receive incentives. By six months, the use of ITNs converged back towards a level similar to the control households. Households which received incentives for ITN use were more likely to use an ITN that they owned after six months than households that did not receive an incentive. Performance-based incentives for behaviour change are a promising tool that may improve the effectiveness of ITN programmes, particularly in achieving immediate coverage of populations most vulnerable to malaria. Incentives offer the opportunity to bypass the gap between ITN ownership and use and put more direct control over who uses ITNs in the hands of policy makers and public health experts. The results of this study demonstrate the potential of incentives to boost ITN use in the immediate term. Further research is needed to determine the longer-term effects of these incentives and the cost-effectiveness of incentives versus traditional education campaigns for boosting the use of ITNs in sub-Saharan Africa.

## Competing interests

The authors declare that they have no competing interests.

## Authors' contributions

PJK and DHH designed the study and created the surveys. PJK and ABC trained the research team and oversaw the fieldwork. PJK and ABC conducted the quantitative and qualitative data analysis. All authors contributed to writing the manuscript, and all read and approved the final manuscript. The opinions expressed are those of the authors and may not reflect the position of their employing organizations or of their funders.

## References

[B1] Lengeler C Insecticide-treated bed nets and curtains for preventing malaria Cochrane Database Syst Rev 2004 2 CD000363 15106149 10.1002/14651858.CD000363.pub2

[B2] Fegan G Noor A Akhwale W Cousens S Snow R Effect of expanded insecticide-treated bednet coverage on child survival in rural Kenya: a longitudinal study Lancet 2008 370 1035 1039 10.1016/S0140-6736(07)61477-9 PMC2117339 17889242

[B3] Chase C Sicuri E Sacoor C Nhalungo D Nhacolo A Alonso PL Menéndez C Determinants of household demand for bed nets in a rural area of southern Mozambique Malar J 2009 8 132 10.1186/1475-2875-8-132 19527505 PMC2706254

[B4] Guyatt H Ochola S Snow R Too poor to pay: charging for insecticide-treated bednets in highland Kenya Trop Med Int Health 2002 7 846 850 10.1046/j.1365-3156.2002.00929.x 12358619

[B5] Webster J Smith L Lines J Scaling-up ITN access and use in sub-Saharan Africa: Estimated LLIN requirements and coverage outcomes based on the global delivery strategy mix 2008 Department for International Development Health Resource Centre

[B6] Wiseman V Scott A McElroy B Conteh L Stevens W Determinants of bed net use in the Gambia: implications for malaria control Am J Trop Med Hyg 2007 76 830 836 17488900

[B7] Minakawa N Dida G Sonye G Futami K Kaneko S Unforeseen misuses of bed nets in fishing villages along Lake Victoria Malar J 2008 7 165 10.1186/1475-2875-7-165 18752662 PMC2532690

[B8] Hopkin M The big push Nature 2008 451 1047 1049 10.1038/4511047a 18305519

[B9] Cohen J Dupas P Free Distribution or Cost-Sharing? Evidence from a Malaria Prevention Experiment NBER Working Paper Series 2008

[B10] Hoffman V Christopher B Just D Do free goods stick to poor households? Experimental evidence on insecticide treated bednets World Development 2009 37 607 617 10.1016/j.worlddev.2008.08.003

[B11] WHO Malaria Vector Control and Personal Protection 2006 http://malaria.who.int/docs/WHO-TRS-936s.pdf WHO Technical Report Series 16623084

[B12] Onwujekwe O Hanson K Fox-Rushby J Inequalities in purchase of mosquito nets and willingness to pay for insecticide-treated nets in Nigeria: Challenges for malaria control interventions Malar J 2004 3 6 10.1186/1475-2875-3-6 15023234 PMC395839

[B13] Maxwell C Rwegoshora R Magesa S Comparison of coverage with insecticide-treated nets in a Tanzanian town and villages where nets and insecticide are either marketed or provided free of charge Malar J 2006 5 44 10.1186/1475-2875-5-44 16712738 PMC1489938

[B14] Kane R Johnson P Town R Butler M A Structured Review of the Effect of Economic Incentives on Consumers' Preventive Behaviour Am J Prev Med 2004 27 327 52 10.1016/j.amepre.2004.07.002 15488364

[B15] Sindelar J Paying for performance: the power of incentives over habits Health Economics 2008 17 449 451 10.1002/hec.1350 18348117 PMC3434678

[B16] Eichler R Can 'Pay for Performance' increase utilization by the poor and improve the quality of health services? 2006 Center for Global Development http://www.cgdev.org/doc/ghprn/PBI%20Background%20Paper.pdf

[B17] Glassman A Todd J Performance-based incentives for health: conditional cash transfer programmes in Latin America and the CaribbeAnopheles 2007 Center for Global Development http://www.cgdev.org/files/13542_file_CCT_LatinAmerica.pdf

[B18] Morris S Olinto P Flores R Nilson E Figueiró A Conditional cash transfers are associated with a small reduction in age-related weight gain in children in Northeast Brazil J Nutrition 2004 134 2336 2341 10.1093/jn/134.9.2336 15333725

[B19] Powell-Jackson T Wolfe R Encouraging women to use professional care at childbirth: Does Nepal's Safe Delivery Incentive Programme work? Evidence from the district of Makwanpur Toward 4+5 2008 http://www.towards4and5.org.uk/PDFs/briefing_paper_2.pdf Briefing Paper

[B20] Beith A Eichler R Weil D Performance-Based Incentives for Health: A way to improve Tuberculosis detection and treatment completion? 2007 Center for Global Development http://www.cgdev.org/files/13544_file_TB_Incentives.pdf Working Paper

[B21] World Bank Country Brief Madagascar 2008

[B22] Molet M Nomenclature des groupes ethniques à Madagascar Bulletin de Madagascar: Travaux de l'Institut de recherche scientifique de Madagascar 1957 129 162 170

[B23] President's Malaria Initiative: Malaria Operational Plan - FY09 http://www.fightingmalaria.gov/countries/mops/fy09/madagascar_mop-fy09.pdf

[B24] World Health Organization (WHO) Mortality Country Fact Sheet 2006 http://www.who.int/whosis/mort/profiles/mort_afro_mdg_madagascar.pdf

[B25] World Health Organization (WHO) Madagascar profile http://www.afro.who.int/malaria/country-profile/madagascar.pdf

[B26] UNICEF Madagascar-Statistics 2008 http://www.unicef.org/infobycountry/madagascar_statistics.html

[B27] Institut Pasteur de Madagascar, Groupe de Recherche sur le Paludisme, Roll Back Malaria Initiative Atlas évolutif du paludisme à Madagascar 2002 http://www.pasteur.mg/spip.php?article286

[B28] MEASURE DHS+ Demographic and Health Survey: Madagascar 2003-2004 http://www.measuredhs.com (accessed 1/15/2010)

[B29] Campbell M Elbourne D Altman D CONSORT statement: extension to cluster randomized trials BMJ 2004 328 702 708 10.1136/bmj.328.7441.702 15031246 PMC381234

[B30] Zou G A Modified Poisson Regression Approach to Prospective Studies with Binary Data Am J Epidemiol 2004 159 702 706 10.1093/aje/kwh090 15033648

[B31] Macintyre K Keating J Okbaldt Y Zerom M Sosler S Ghebremeskel T Eisele T Rolling out insecticide treated nets in Eritrea: examining the determinants of possession and use in malarious zones during the rainy season Trop Med Int Health 2006 11 824 233 10.1111/j.1365-3156.2006.01637.x 16772004

[B32] Frey C Traoré C De Allegri M Kouyaté B Müller O Compliance of young children with ITN protection in rural Burkina Faso Malar J 2006 5 70 10.1186/1475-2875-5-70 16907964 PMC1570361

[B33] Toé L Skovmand O Dabiré K Diabaté A Diallo Y Guiguemdé T Doannio J Akogbeto M Baldet T Gruénais M Decreased motivation in the use of insecticide-treated nets in a malaria endemic area in Burkina Faso Malar J 2009 8 175 10.1186/1475-2875-8-175 19640290 PMC2729312

[B34] Deci E Ryan R Intrinsic motivation and self-determination in human behaviour 1985 New York: Plenum Press

[B35] Melnikow J Paliescheskey M Stewart G Effect of a transportation incentive on compliance with the first prenatal appointment: a randomized trial Obstet Gynecol 1997 89 1023 7 10.1016/S0029-7844(97)00147-6 9170485

[B36] Marcus A Crane L Kaplan C Reading AE Savage E Gunning J Bernstein G Berek JS Improving adherence to screening follow-up among women with abnormal Pap smears: results from a large clinic-based trial of three intervention strategies Med Care 1992 30 216 30 10.1097/00005650-199203000-00004 1538610

[B37] Baume C Marin MC Gains in awareness, ownership and use of insecticide-treated nets in Nigeria, Senegal, Uganda and Zambia Malar J 2008 7 153 10.1186/1475-2875-7-153 18687145 PMC2527013

[B38] Alliance for Malaria Prevention(AMP) A toolkit for developing integrated campaigns to encourage the distribution and use of long lasting insecticide-treated nets 2008 http://www.aed.org/Publications/upload/LLIN_English.pdf

